# Cabergoline Therapy and Tumor Growth Rate in Pituitary Microadenomas: A Retrospective Cohort Study

**DOI:** 10.3390/jcm15052054

**Published:** 2026-03-08

**Authors:** Abdurrahim Tekin, Engin Can, Evren Sönmez, Lokman Ayhan, Suna Dilbaz, Akın Öztürk, Enis Furkan Edehan, Serdar Çevik, Nuri Serdar Baş

**Affiliations:** Department of Neurosurgery, Kanuni Sultan Süleyman Training and Research Hospital, 34303 Istanbul, Türkiye

**Keywords:** pituitary microadenoma, hyperprolactinemia, cabergoline, tumor growth rate, dopamine agonist, prolactin, MRI

## Abstract

**Objective**: To compare tumor growth rate between patients with pituitary microadenomas who had mild to moderate prolactin elevation and symptoms leading to initiation of cabergoline therapy, and asymptomatic microadenomas without prolactin elevation managed with observation. **Materials and Methods**: In this retrospective cohort study, 139 patients diagnosed with pituitary microadenoma between 2019 and 2024 and with at least 12 months of clinical and radiological follow-up were included. Patients who received cabergoline therapy due to symptoms were classified as the dopamine agonist-positive [DA(+)] group, while those who did not receive treatment were classified as the dopamine agonist-negative [DA(−)] group. Tumor growth rate was calculated as the annual change (mm/year) in maximum tumor diameter on serial magnetic resonance imaging. Between-group comparisons were performed using the Mann–Whitney U test. A mixed-effects linear model was constructed to evaluate the interaction between time and treatment. **Results**: Of the 139 patients included in the study, 42 were in the DA(+) group and 97 were in the DA(−) group. There were no significant differences between the groups in terms of baseline age, follow-up duration, or tumor size (*p* > 0.05). The mean tumor growth rate was 0.67 ± 0.80 mm/year in the DA(−) group and 0.36 ± 0.38 mm/year in the DA(+) group (*p* = 0.0208). In the mixed-effects model analysis, the time × treatment interaction was statistically significant (β = −0.021 mm/month; *p* = 0.009). Patients receiving cabergoline showed a marked reduction in prolactin levels and improvement in symptoms in 78% of cases. Importantly, no tumor shrinkage was observed in either group; the primary observed effect was a reduction in growth velocity rather than true tumor regression. No serious treatment-related adverse effects were observed. **Conclusions**: In patients with pituitary microadenomas, cabergoline therapy was associated with a reduced tumor growth rate over time, while no true tumor regression was observed. These findings suggest that cabergoline exposure may influence longitudinal tumor growth dynamics in clinically ambiguous cases encountered in routine practice, without implying definitive tumor subtype classification.

## 1. Introduction

The pituitary gland is the central regulator of the endocrine system and is responsible for the synthesis, storage, and secretion of numerous hormones. Pituitary tumors are among the most common intracranial neoplasms in adulthood and are referred to as pituitary neuroendocrine tumors (PitNETs) in current classifications [1]. These tumors are classified according to size as microadenomas (<10 mm) and macroadenomas (≥10 mm), and according to functional status as functioning (actively hormone-secreting) and non-functioning (without biochemically evident hormone hypersecretion) [1,2]. Functioning adenomas present with syndromes of hormonal excess (e.g., prolactinoma, acromegaly, Cushing disease), whereas non-functioning adenomas are more commonly diagnosed due to mass effect or signs of hypopituitarism. With the widespread use of pituitary imaging, more frequently detected are pituitary incidentalomas, a substantial proportion of which are microadenomas [1,3].

Longitudinal data indicate that most pituitary microadenomas follow an indolent course; however, a subset demonstrates measurable growth over time, with estimated annual growth rates of approximately 1–2 cases per 100 person-years and a 5-year cumulative probability of growth of approximately 14–15% in large cohort studies [3,4]. Tumor growth in macroadenomas may lead to clinically significant consequences, such as optic chiasm compression, visual impairment, and pituitary insufficiency, whereas microadenomas are most often detected incidentally and tend to follow a more indolent course [3,5]. Therefore, the primary clinical question in microadenomas is whether tumor growth occurs during long-term follow-up and which factors influence growth dynamics.

In clinical practice, diagnostic differentiation is not always clear in pituitary microadenomas detected in association with mild to moderate hyperprolactinemia. Prolactinomas account for approximately 40–50% of functioning pituitary adenomas; however, mild hyperprolactinemia in patients with microadenomas may also reflect the stalk effect or other etiologies, particularly when prolactin levels remain below 100–200 ng/mL [6,7,8]. On the other hand, small prolactin-secreting adenomas (microprolactinomas) may also present with low to moderate prolactin levels. Accordingly, after exclusion of recognized secondary causes, a subset of patients remains in whom the etiological distinction between microprolactinoma and non-functioning adenoma cannot be definitively established in routine practice, representing a clinically ambiguous group.

In such clinically ambiguous cases, treatment decisions are often guided by symptoms rather than definitive diagnosis. In the presence of symptoms related to hyperprolactinemia—such as amenorrhea, galactorrhea, infertility, and decreased libido—dopamine agonists like cabergoline are widely used in clinical practice [9]. Although cabergoline can achieve a rapid reduction in prolactin levels and symptomatic improvement, a marked decrease in tumor size is not always expected in this heterogeneous group of microadenomas [6,9]. Nevertheless, whether dopamine agonists exert effects beyond biochemical and clinical relief—specifically on tumor growth dynamics—remains insufficiently clear, particularly at the microadenoma level.

The primary approach to the management of non-functioning adenomas involves individualized surveillance and, when necessary, surgical or radiotherapy options based on tumor size, symptom presence, and growth tendency [1,3]. While medical therapy is standardized for functioning tumors, dopamine agonists are not routinely recommended for direct tumor control in non-functioning adenomas; however, they are commonly used in clinical practice for the management of symptomatic hyperprolactinemia [9]. Therefore, an objective assessment of tumor growth rate in this clinically ambiguous group of microadenomas receiving cabergoline may contribute to a more rational determination of surveillance strategies.

The aim of this study was to compare tumor growth rate between patients with pituitary microadenomas presenting with mild to moderate prolactin elevation and symptoms prompting cabergoline therapy, and those with asymptomatic microadenomas without prolactin elevation who were managed conservatively. Without claiming definitive diagnostic classification, the study aims to clarify the relationship between cabergoline exposure and tumor growth dynamics in this clinically ambiguous population encountered in real-world clinical practice.

## 2. Materials and Methods

### 2.1. Study Design and Patient Selection

This retrospective cohort study included patients aged 18–60 years diagnosed with pituitary microadenoma at a tertiary care training and research hospital between January 2019 and December 2024 who had at least 12 months of radiological follow-up.The inclusion criteria were as follows:

- Maximum adenoma diameter of less than 10 mm on magnetic resonance imaging (MRI);
- Availability of at least 12 months of follow-up from the time of diagnosis, including follow-up MRI examinations;
- Other anterior pituitary hormones (TSH, ACTH, cortisol, LH, FSH, IGF-1) being within normal reference ranges in accordance with clinical evaluation;
- Patients with mild to moderate prolactin elevation (as defined below) accompanied by symptoms (amenorrhea, galactorrhea, infertility, decreased libido) in whom cabergoline therapy was initiated for symptom control;
- Asymptomatic patients with pituitary microadenomas without prolactin elevation who were managed with observation.

### 2.2. Exclusion Criteria

Patients with macroadenomas (≥10 mm), functioning pituitary tumors, prior pituitary surgery or radiotherapy were excluded. Patients with marked hyperprolactinemia (>200 ng/mL) suggestive of typical prolactinoma were excluded. Secondary causes of hyperprolactinemia—including pregnancy, lactation, oral contraceptive use, dopamine antagonist medications, primary hypothyroidism, chronic renal failure, and clinically evident polycystic ovary syndrome—were systematically evaluated and excluded prior to inclusion. Active pregnancy during diagnosis or follow-up was an exclusion criterion. Patients unable to tolerate cabergoline due to adverse effects or demonstrating non-adherence were excluded.

### 2.3. Radiological Assessment

All pituitary MRI examinations were performed using a 1.5 Tesla Philips Ingenia scanner (Philips Medical Systems, Best, The Netherlands). Contrast-enhanced coronal and sagittal T1-weighted sequences were used. Measurements were independently performed by two experienced observers who were blinded to clinical information. In each examination, the maximum tumor diameter (mm) was measured and recorded. To minimize potential technical variability between measurements, the same imaging plane and sequence parameters were used. Tumor growth tendency was assessed by calculating the difference in diameter between the initial and final MRI scans for each patient. Individual tumor growth rate was defined as follows:

Tumor growth rate (mm/year) = (Final diameter (mm) − Initial diameter (mm))/Duration of follow-up (years)

### 2.4. Endocrinological Assessment and Cabergoline Therapy

Patients in whom cabergoline therapy was initiated due to clinical symptoms (amenorrhea, galactorrhea, infertility, decreased libido, etc.) constituted the treatment group of the study. In these patients, cabergoline therapy was administered for the control of hyperprolactinemia-related symptoms and to achieve biochemical improvement, and was not intended as a tumor-reducing or antitumor treatment.

Cabergoline therapy was initiated in accordance with protocols generally accepted in the literature. The starting dose was 0.25 mg twice weekly and was increased to 0.5 mg twice weekly based on symptomatic and biochemical response. When necessary, the dose was titrated within a range of 0.25–2.0 mg/week according to tolerability. Dopamine agonist-related adverse effects, such as dizziness, orthostatic hypotension, nausea, or gastrointestinal discomfort, were monitored. Patients who were unable to adhere to treatment or who discontinued therapy due to adverse effects were excluded from the study.

Patients receiving cabergoline were defined as the DA(+) group, while those who did not receive any treatment were defined as the DA(−) group. Changes in tumor diameter and tumor growth rates were compared between the two groups.

### 2.5. Data Collection

Patient age, sex, dates of initial and final MRI examinations, prolactin levels, other pituitary hormone results, and cabergoline dose and duration were extracted from medical records. The institutional laboratory reference ranges for prolactin were 4.6–21.4 ng/mL for men and 6.0–29.9 ng/mL for women. Mild hyperprolactinemia was defined as values above the sex-specific upper reference limit but <100 ng/mL, and moderate hyperprolactinemia as 100–200 ng/mL. Tumor growth rate (mm/year), calculated from the change in tumor diameter over time, was used as the primary outcome variable in the analysis.

## 3. Statistical Analysis

Using all MRI measurements available for each patient, an individual linear growth slope (mm/month) was calculated. These slopes were compared between patients receiving cabergoline (DA+) and those not receiving cabergoline (DA−) using the Mann–Whitney U test. In addition, a mixed-effects linear model was constructed to evaluate the interaction between time and cabergoline treatment. In this model, time (months), group (DA+/DA−), and the time × group interaction were specified as fixed effects, while patient-level random intercepts and slopes (1 + time | patient) were included as random effects. Age, sex, baseline tumor diameter (mm), and follow-up duration (months) were included in the model as covariates.

Statistical analyses were performed using IBM SPSS Statistics version 26.0 (IBM Corp., Armonk, NY, USA) and R software version 4.3.1 (R Foundation for Statistical Computing, Vienna, Austria); the mixed-effects model analysis was conducted using the lme4 package (lmer function). Normality of continuous variables was assessed using the Shapiro–Wilk test. Variables that did not follow a normal distribution were presented as median [interquartile range (IQR), 25th–75th percentile]. Model results were reported as β coefficients with 95% confidence intervals (CIs). Continuous variables were presented as mean ± standard deviation (SD) or median [interquartile range, IQR], and categorical variables as number (percentage). Comparisons between two independent groups were performed using the Mann–Whitney U test for non-normally distributed variables. A two-sided *p* value < 0.05 was considered statistically significant.

Because the study was designed retrospectively, a priori power analysis was not performed. However, the sample size (*n* = 139) was considered adequate compared with similar studies, and post hoc calculations indicated that the study had 80% power to detect a difference in tumor growth rate of 0.4 mm/year at an α level of 0.05.

## 4. Ethics Approval

The study was conducted in accordance with the Declaration of Helsinki and approved by the Ethics Committee of Kanuni Sultan Süleyman Training and Research Hospital (protocol code 2025.11.327; approved on 28 November 2025).

## 5. Results

### 5.1. Study Flow

**Figure 1:**
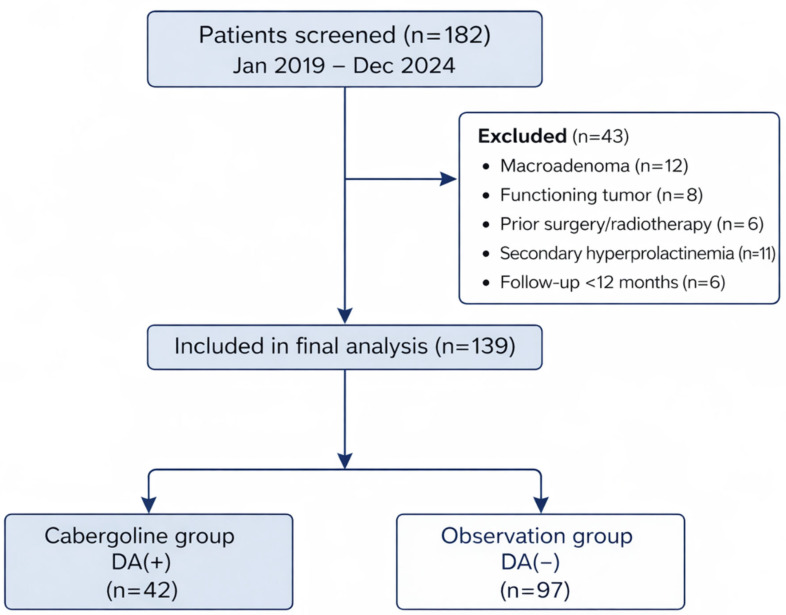
Flow diagram of patient selection and group allocation.

A total of 182 patients were screened between January 2019 and December 2024. After applying exclusion criteria, 139 patients met the inclusion criteria and were included in the final analysis (Figure 1).

### 5.2. Patient Characteristics

A total of 139 patients were included in the study. Of these, 42 patients received cabergoline therapy (DA+) due to clinical symptoms, while 97 asymptomatic patients without prolactin elevation were managed with observation alone (DA−).

Overall, 76% of the patients were female (*n* = 106) and 24% were male (*n* = 33). The proportion of women was higher in the cabergoline-treated group; among the 42 patients in the DA(+) group, 37 were female and 5 were male. The mean age was similar between the two groups (DA(+): 35.1 ± 9.1 years; DA(−): 37.1 ± 11.7 years; *p* = 0.281).

The mean follow-up duration was 22.9 ± 12.0 months in the DA(+) group and 20.9 ± 10.4 months in the DA(−) group. Median (interquartile range) follow-up durations were 24 (12–33) months and 18 (12–24) months, respectively. There was no significant difference between the groups in terms of baseline tumor diameter (DA(+): 4.29 ± 1.75 mm; DA(−): 4.47 ± 1.93 mm; *p* = 0.596) (Table 1).

### 5.3. Baseline Prolactin Levels

Baseline (month 0) prolactin levels differed between the groups, consistent with the clinical indications for initiating cabergoline therapy:

DA(−) group: 18.9 ± 16.5 ng/mL; median 15.0 [9.0–22.0] ng/mL

DA(+) group: 66.0 ± 26.1 ng/mL; median 58.5 [47.3–86.3] ng/mL

In patients receiving cabergoline therapy, prolactin levels remained within the mild to moderate range, and the decision to initiate treatment was based on the presence of clinical symptoms.

### 5.4. Tumor Growth Rate

At least two MRI measurements obtained at different time points were used for each patient to calculate individual tumor growth rate.

**Figure 2:**
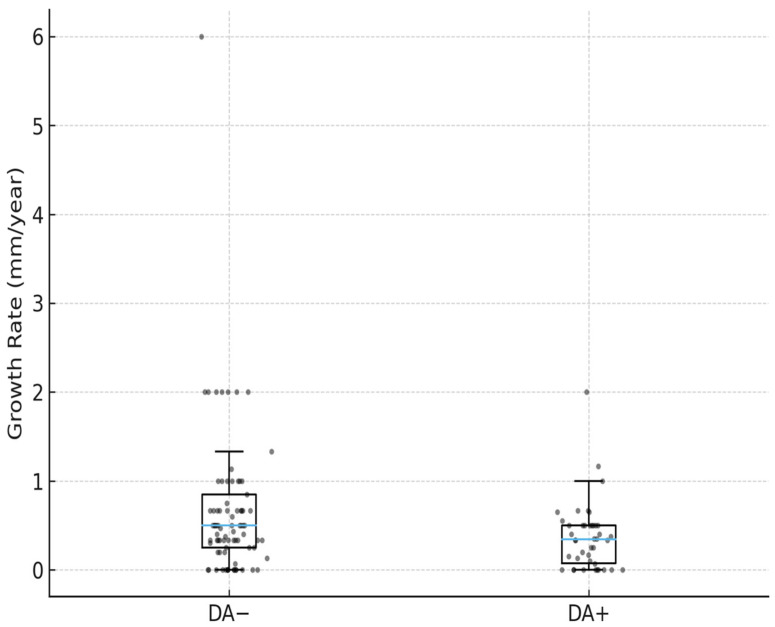
Comparison of annual tumor growth rates between DA(–) and DA(+) groups. Gray dots represent individual patient measurements, and the blue boxplots represent the distribution of tumor growth rates within each group.

According to the results, tumor growth rate was found to be significantly lower in patients receiving cabergoline therapy:

DA(−) group: Mean growth rate 0.67 ± 0.80 mm/year (95% CI: 0.51–0.83), median 0.50 [0.25–0.85] mm/year;

DA(+) group: Mean growth rate 0.36 ± 0.38 mm/year (95% CI: 0.24–0.48), median 0.35 [0.08–0.50] mm/year;

The difference between the two groups was statistically significant (Mann–Whitney U = 2541.5; *p* = 0.0208). The between-group difference in tumor growth rate ranged from −0.59 to −0.04 mm/year (95% CI) (Figure 2).

No radiologically confirmed tumor shrinkage was observed in either group during follow-up.

### 5.5. Mixed-Effects Model Analysis

A mixed-effects linear model was constructed using all diameter measurements obtained over time (0–48 months). In the model, time (months), cabergoline therapy, and the time × treatment interaction were specified as fixed effects, while patient-level random intercepts and slopes (1 + time | patient) were defined as random effects. Age and baseline tumor diameter were included in the model as covariates.

**Figure 3:**
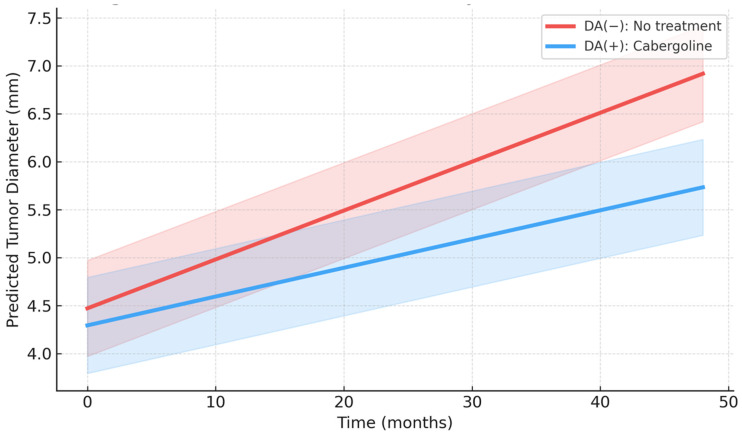
Predicted tumor diameter trajectories over time according to treatment group.

The mixed-effects model showed a significant effect of time (β = +0.051 mm/month; 95% CI 0.032–0.070; *p* < 0.001). Cabergoline therapy itself was not significantly associated with tumor diameter (β = −0.031 mm; 95% CI −0.122–0.060; *p* = 0.492). However, the time × treatment interaction was statistically significant (β = −0.021 mm/month; 95% CI −0.037 to −0.005; *p* = 0.009). Baseline tumor diameter was strongly associated with tumor size over time (β = +1.01 mm; 95% CI 0.92–1.09; *p* < 0.001), whereas age showed no significant association (β = +0.0004; 95% CI −0.018–0.019; *p* = 0.989). This corresponds to an approximately 0.25 mm/year slower increase in tumor diameter in the cabergoline-treated group (Table 2, Figure 3).

### 5.6. Clinical Observations and Additional Findings

In patients receiving cabergoline therapy, the mean pre-treatment prolactin level was 66.0 ± 26.1 ng/mL, which decreased to a mean of 26 ± 15 ng/mL by the end of the first year of treatment. Marked improvement in hyperprolactinemia-related clinical symptoms was observed in 78% of cases, while symptomatic improvement was limited in 22%.

Mild treatment-related adverse effects (nausea, dizziness, orthostatic hypotension) were observed in 4 patients (9.5%) and were controlled with dose reduction; no serious adverse events were identified (Table 3).

In the DA(−) group, prolactin levels remained within normal reference ranges throughout follow-up. In this group, a marked increase in tumor diameter (>1 mm/year) was observed in approximately one-third of tumors during long-term follow-up.

## 6. Discussion

In this study, we evaluated tumor growth dynamics in patients with pituitary microadenomas according to exposure to cabergoline therapy in routine clinical practice. Our findings demonstrate that patients receiving cabergoline therapy exhibited a significantly lower rate of tumor diameter increase, and that the time × treatment interaction remained independently significant in the mixed-effects model. No radiologically confirmed tumor regression was observed in either group; rather, the principal effect identified was a slowing of tumor growth.

Large-scale studies examining the natural history of non-functioning pituitary adenomas (NFPAs) have shown that the vast majority of microadenomas remain stable during long-term follow-up, although progressive growth may occur in a specific subgroup. In a comprehensive review published by Yavropoulou and colleagues, it was emphasized that although the annual growth rate of microadenomas is low, it cannot be completely disregarded [2]. Similarly, in the systematic review by Rikvold et al., the growth rate of microadenomas was reported to be approximately 1–2 per 100 person-years, and conservative surveillance was considered safe for most patients [3]. In our study, the mean growth rate observed in the untreated group (0.67 mm/year) was consistent with these natural history data.

In contrast, the significantly lower growth rate observed in the cabergoline-treated group suggests that dopamine agonists may be capable of modulating tumor dynamics in this patient population. The potential effect of dopamine agonists in NFPAs has attracted increasing interest in recent years. In a systematic review and meta-analysis published by Pivonello et al. in 2022, tumor stabilization or shrinkage was reported in a substantial proportion of NFPA patients treated with cabergoline [1]. Likewise, in the prospective study by Vargas-Ortega and colleagues, significant tumor shrinkage was observed in 66% of cabergoline-treated NFPA patients, stable disease in 25%, and progression in only 9% [10]. Although these studies predominantly included macroadenomas and heterogeneous patient populations, they indicate that dopamine agonists are not biologically ineffective in NFPAs [10,11].

The absence of marked tumor regression observed in our study may be explained by the fact that the study population consisted of microadenomas and represented a clinically and diagnostically uncertain subgroup. Kawaguchi and colleagues reported that patients with prolactin levels in the range of 90–200 ng/mL constitute the most diagnostically challenging group and that, in these patients, dopamine agonists may reduce prolactin levels without consistently inducing tumor volume reduction [6]. In this context, the slowing of tumor growth rate without overt shrinkage observed in our study may be considered a clinically meaningful finding that differs from the classic response pattern seen in prolactinomas.

The biological basis of the potential antiproliferative effect of dopamine agonists is related to dopamine D2 receptor expression in pituitary tumors. In the meta-analysis and review published by Wexler and Page-Wilson, D2 receptors were shown to be expressed at variable densities not only in prolactinomas but also across multiple PitNET subtypes, including non-functioning adenomas [9]. Activation of D2 receptors may result in inhibition of the adenylate cyclase pathway and suppression of cellular proliferation. However, expression of these receptors is heterogeneous in non-functioning adenomas, which may explain the variable response to dopamine agonists observed in this group.

In clinical practice, the primary indication for cabergoline therapy in this patient population is usually the control of symptoms related to hyperprolactinemia. The “cabergoline disconnection test”, described by Galliano and colleagues, has been proposed as a tool through which biochemical response to dopamine agonists may provide indirect information about tumor biology [12]. Although this test is not a diagnostic standard, it supports the concept that dopamine responsiveness may be associated with tumor behavior. In our study, the observation of an average reduction of more than 60% in prolactin levels and symptomatic improvement in 78% of patients receiving cabergoline is consistent with the clinical benefits reported by Pérez-López et al. and Majumdar and Mangal [8,13].

The high proportion of women in the cabergoline-treated group (88%) in our study is consistent with the epidemiological distribution reported in the literature. Whyte and colleagues reported that pituitary microadenomas in women are more frequently detected at an earlier stage, often due to symptoms related to reproductive function [7]. The modulatory effect of estrogen on prolactin secretion may also contribute to this sex difference [13].

One of the most recent and comprehensive datasets on the natural history of non-functioning pituitary microadenomas comes from the UK NFPA Consortium study by Hamblin and colleagues. In that study, the 5-year cumulative growth rate among 459 microadenoma cases was reported to be 14.5%, and it was suggested that intervals during the follow-up could be extended [4]. In our study, growth rates observed in the untreated group were consistent with this natural history, whereas the marked slowing observed in the cabergoline-treated group suggests that dopamine agonists may confer an additional advantage that could influence surveillance strategies.

The safety profile of cabergoline was also favorable in our cohort. As reported by Wexler and Page-Wilson, cabergoline is generally well-tolerated at low to moderate doses during long-term use, and serious cardiac adverse effects occur only at very high cumulative doses [9]. In our series, no serious adverse events were observed, and only mild and transient adverse effects were noted in 9.5% of patients. This rate is consistent with the safety data reported by Capatina and Poiana [14].

Several limitations of our study should be acknowledged. These include the retrospective design, the use of maximum diameter rather than tumor volume for assessment, and heterogeneity in prolactin measurement methods. In addition, although recognized secondary causes of hyperprolactinemia were systematically excluded, subclinical or multifactorial conditions that may influence prolactin levels or mimic hyperprolactinemia-related symptoms cannot be entirely ruled out due to the retrospective nature of the study, and this may represent a potential source of residual confounding. Nevertheless, the use of a mixed-effects model allowed for control of time-dependent variables and partially reduced the impact of individual variability. Future prospective, long-term studies are needed to more clearly define the role of cabergoline in this diagnostically uncertain population.

## 7. Conclusions

In this retrospective cohort study, tumor growth dynamics were analyzed according to exposure to cabergoline therapy in patients with pituitary microadenomas managed in routine clinical practice. The findings demonstrate that patients receiving cabergoline therapy exhibited a significantly reduced rate of tumor diameter increase, and that this effect remained independently significant over time. While cabergoline therapy resulted in marked reductions in prolactin levels and symptomatic improvement, a pattern of slowed growth or stabilization—rather than typical tumor shrinkage—was observed in this patient population.

In these clinically ambiguous cases, where definitive diagnostic classification cannot always be established, cabergoline therapy may be considered a supportive option within surveillance strategies, primarily for symptomatic control and potentially for modulation of tumor growth dynamics. These findings require confirmation through prospective studies with longer-term follow-up.

## Figures and Tables

**Table 1:** Baseline Characteristics of the Study Population.

Variable	DA(+) (*n* = 42)	DA(−) (*n* = 97)	*p* Value
Age (years), mean ± SD	35.1 ± 9.1	37.1 ± 11.7	0.281
Female sex, *n* (%)	37 (88%)	69 (71%)	0.032
Baseline tumor diameter (mm), mean ± SD	4.29 ± 1.75	4.47 ± 1.93	0.596
Follow-up duration (months), mean ± SD	22.9 ± 12.0	20.9 ± 10.4	0.364
Baseline prolactin (ng/mL), median [IQR]	58.5 [47.3–86.3]	15.0 [9.0–22.0]	<0.001

DA(+), cabergoline-treated group; DA(−), untreated group; SD, standard deviation; IQR, interquartile range.

**Table 2:** Tumor Growth Outcomes and Mixed-Effects Model Results.

Outcome/Parameter	DA(+) (*n* = 42)	DA(−) (*n* = 97)	Effect Size/95% CI
Tumor growth rate (mm/year), mean ± SD	0.36 ± 0.38	0.67 ± 0.80	Δ −0.31 (95% CI −0.59 to −0.04)
No tumor growth, *n* (%)	36 (85%)	42 (43%)	—
Significant growth (>1 mm/year), *n* (%)	6 (15%)	33 (34%)	—
Time × treatment interaction (mm/month)	—	—	β −0.021 (95% CI −0.037 to −0.005)
Final tumor diameter (mm), mean ± SD	4.96 ± 1.93	5.51 ± 2.21	—

DA(+), cabergoline-treated group; DA(−), untreated group; CI, confidence interval; β, regression coefficient.

**Table 3:** Treatment-Related Adverse Events.

Adverse Event	DA(+) (*n* = 42)	DA(−) (*n* = 97)
Nausea	2 (4.8%)	0
Dizziness	1 (2.4%)	0
Orthostatic hypotension	1 (2.4%)	0
Serious adverse events	0	0

No adverse events were observed in the DA(−) group, as no dopamine agonist therapy was administered.

## Data Availability

The data presented in this study are available from the corresponding author upon reasonable request.
